# Measurement of external rotation of the shoulder in patients with obstetric brachial plexus palsy

**DOI:** 10.1186/1749-7221-7-8

**Published:** 2012-10-15

**Authors:** Gerhard Blaauw, Robert S Muhlig

**Affiliations:** 1 Department of Neurosurgery, University Hospital Maastricht, P.O.Box 5800, 6202 AZ, Maastricht, The Netherlands; 2 Rehabilitation Out-Patient Clinic, R.A.P., Parallelweg 2, 6411 ND, Heerlen, The Netherlands

## Abstract

A discussion is presented concerning scoring while assessing shoulder function. Divergence in observation and in interpretation of what is observed may give rise to serious disagreement about indications for surgery. Agreement regarding starting points of measurement is essential. One must realize that the number of degrees, obtained using a scoring system, may not reflect the real *amount* of motion *per se*, it may solely indicate the *limit* of the motion in relation to the neutral zero point of the measurement. This realization may improve the justification of and the indication for surgical treatment.

It is worthy of mention that this paper deals in particular with **active** external rotation. Indications for secondary surgery to prevent gleno-humeral deformation if passive external rotation is diminishing progressively, is a separate topic. We wish to point out that the insidious problem of fixed deformity, even to a minor degree, will contribute to the problem of loss of active functional movement.

## Introduction

The lack of full active external rotation of the shoulder joint is presented in many publications as an indication for neuro-reconstructive surgery (specifically, transfer of the accessory nerve to the suprascapular nerve) in cases of obstetric brachial plexus palsy [1]. Thus the correctness of the measurement of the external rotation and the interpretation of the results is crucial. Although the infraspinatus muscle innervated by the suprascapular nerve, is regarded as the main external rotator of the shoulder joint, external rotation can also be achieved by thoraco-scapular movements. The Mallet score [2], in particular the modification by Tassin [3], is (one of several methods) often used to express the degree of external rotation. This method scores range of motion and composite movement and not individual muscular motion, so that even in cases of complete ankylosis of the gleno-humeral joint, it is possible to obtain a satisfying score because of sufficient thoraco-scapular motion. Another aspect of using scores is the possibility of divergence of opinion between observers, due to misinterpretation of the measurement results.

This problem is not new as illustrated by the following citation. In 1920, Clark [4] from the Mayo Clinic remarked: “When an angle is specifically stated in degrees the reader is sometimes at a loss to know which angle is meant, whether it is the angle included between the two bones concerned in deformity or movement, or the angle between one of the bones and an imaginary line projected into space from the bones”. Measuring and recording joint motion is a matter of agreement. If the measurement results are regarded as absolute facts, this may lead to an incorrect decision on treatment. Thus measurement results must be converted to simple muscular movements and distinguished from global movements.

In 1936, Cave and Roberts [5], from the Committee on Joint Measurement, presented a standard system for measuring and recording joint function. It was the result of a ten years’ trial, plus suggestions from members of the American College of Surgeons. The first of five general principles was “all motions should be measured by degrees from a neutral point of zero” and the second: “the neutral point from which the motion is measured must be defined”. Their definition of the zero neutral point differed considerably from Clark’s system. The internationally approved publication: *Joint Motion. Method of measuring and recording* published in 1965 [6], was based on the principles of Cave and Roberts’ Neutral Zero Method. This contains the same problem as sketched above: joint motion does not usually reflect movement caused by one muscle and is usually the result of several muscles acting, and in the case of the shoulder, of action in more than one joint.

### Rotation of the shoulder

**Figure 1:**
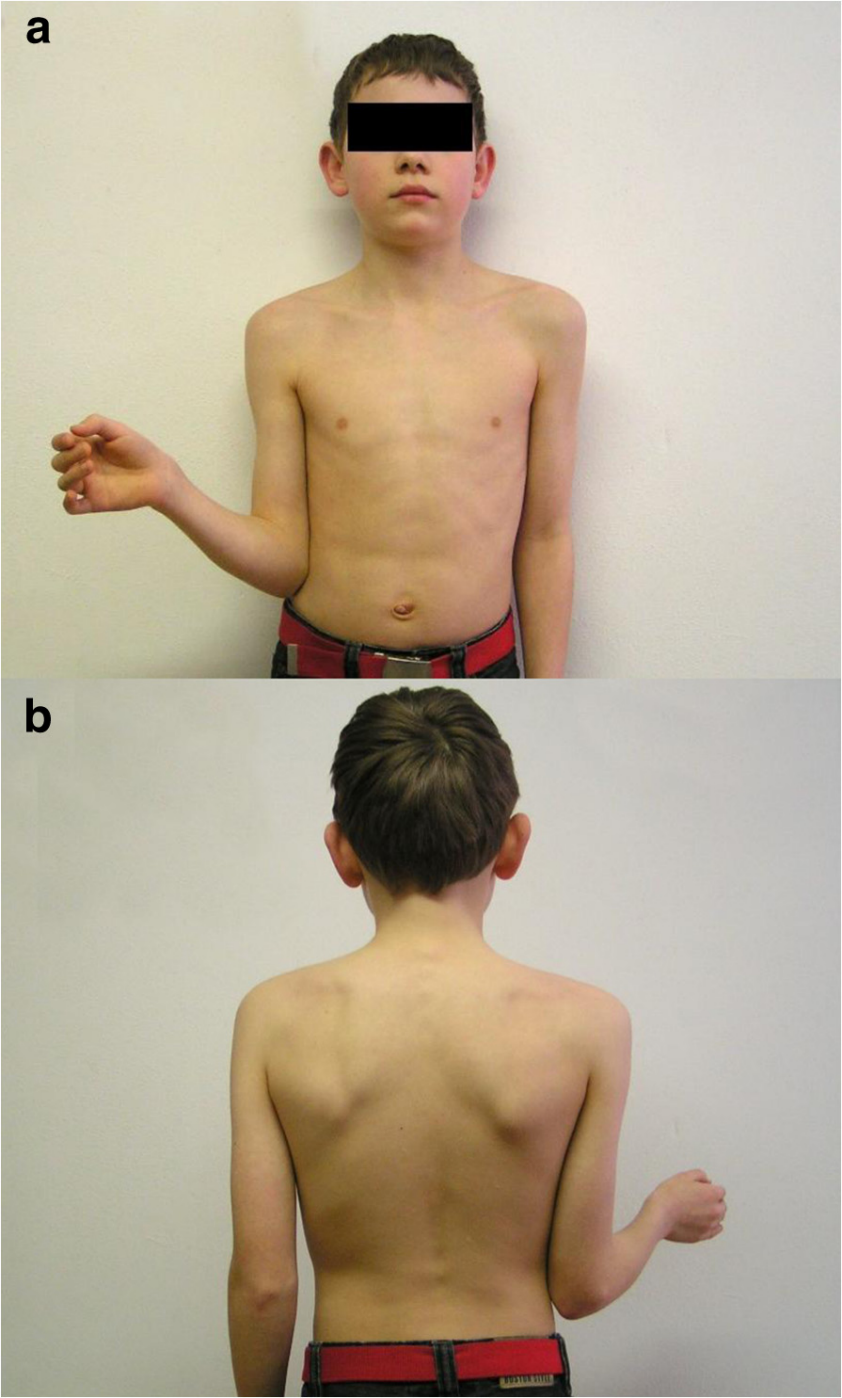
External rotation with the scapula not participating: glenohumeral rotation, 1b External rotation with the scapula not participating: glenohumeral rotation.

**Figure 2:**
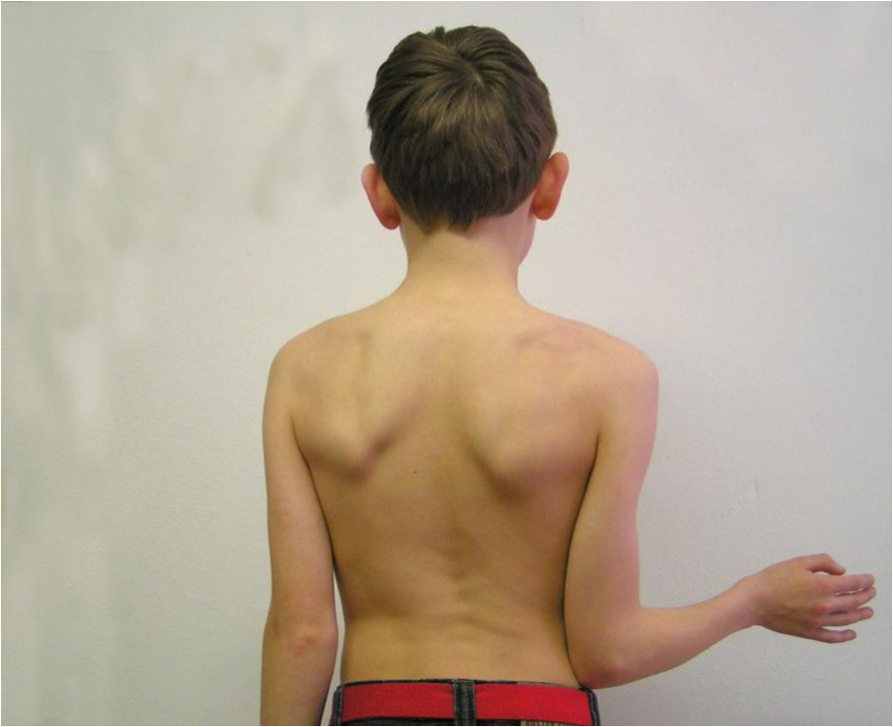
External rotation with glenohumeral movement complemented by thoraco-scapular movement: functional external rotation.

When investigating the limits of rotation in the shoulder articulation, one must realize that the scapula moves, to greater or lesser extent, with all the movements of the humerus. It is, therefore, necessary to try to immobilize the scapula while determining the range of rotation of the shoulder. In this way, gleno-humeral external rotation (Figure 1a and b) can be distinguished from functional external rotation (Figure 2), which is executed by combined thoraco-scapular and gleno-humeral movements. Thus external rotation with the arm held in stable adduction reflects gleno-humeral external rotation which is mainly exerted by the infraspinatus muscle. If stable adduction is not created, external rotation is achieved by a combination of muscles and joints and is thus called functional external rotation.

**Figure 3:**
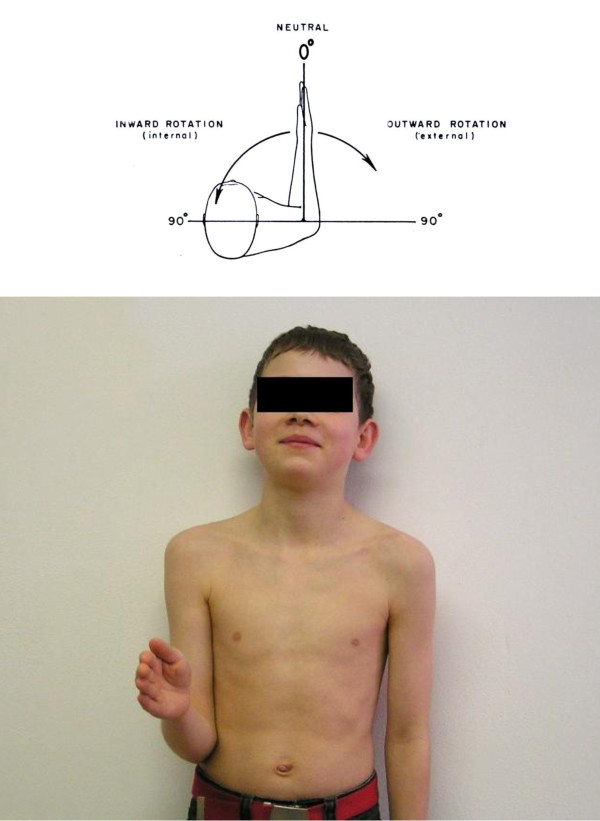
**Rotation with arm at side (from: Joint Motion). **3b External rotation with the arm at side to the neutral zero position in an individual.

**Figure 4:**
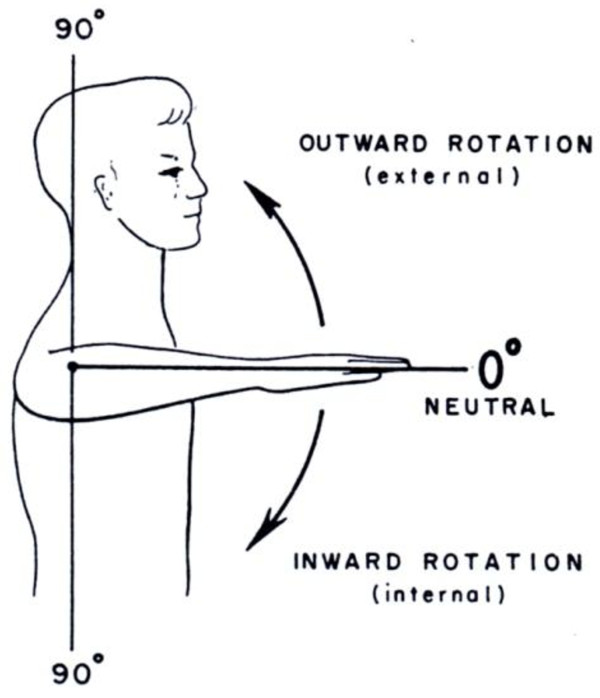
Rotation in abduction (from: Joint Motion).

**Figure 5:**
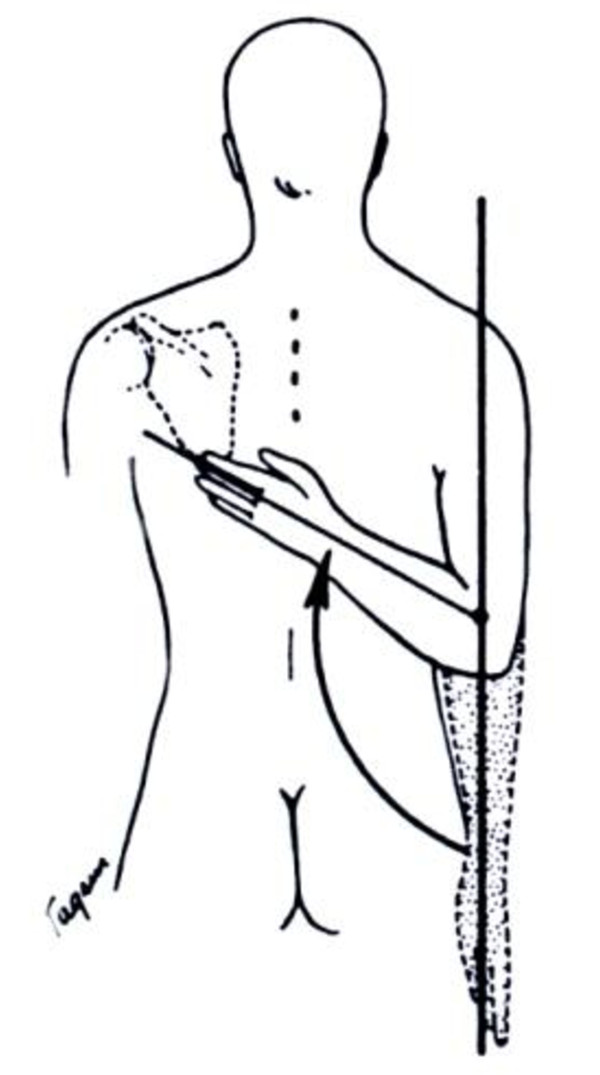
Internal rotation posteriorly (from: Joint Motion).

Passive and active rotation can be measured in adduction or abduction. First with the arm in adduction (arm at the side of the body) with the elbow flexed 90° and the forearm pointing forward. This is called the neutral position: the 0° position (Figure 3a and b). Secondly the rotation can be measured with the arm in 90° abduction, 90° elbow flexion: de arm is held horizontally. This position is then also called the neutral or 0° position (Figure 4). The advantage of the second neutral position is that rotation in this position is pure gleno-humeral in a more restricted sense; the disadvantage is that the muscles holding the shoulder, in particular the scapula, must function normally. Thus for the measurement of active and passive rotation in patients with brachial plexus lesions, measurement in adduction is usually chosen; on the other hand, guidelines from the American Medical Association for evaluating impairment recommend measuring with the shoulder in 90° abduction [7,8]. Furthermore, the limits of rotation in abduction and adduction differ inter- and intra-individually. Testing extreme internal rotation is performed with the arm behind the back (Figure 5). 

Expressing the range of active rotation must be performed with caution. While testing the passive mobility, the examiner tries to put the arm against the patient’s abdomen.

This is the −70° position. Secondly, the examiner rotates the arm with fixed scapula as far externally as possible, which is normally +70°. This means that with this test the passive mobility is 140°. When we test the active motion by asking the patient to rotate from the position hand–to-abdomen to the neutral 0° position, he has actually externally rotated 70°; when he then is able to rotate to, for example, the +20° position, his total range of external rotation is 90° and not 20°. Vice versa, when rotating his arm from the +20° position to the −70° position, he has rotated 90° internally. Thus the number of degrees indicates the *limit* of motion in relation to the neutral zero point and not the *amount* of the motion.

### The Mallet score

**Figure 6:**
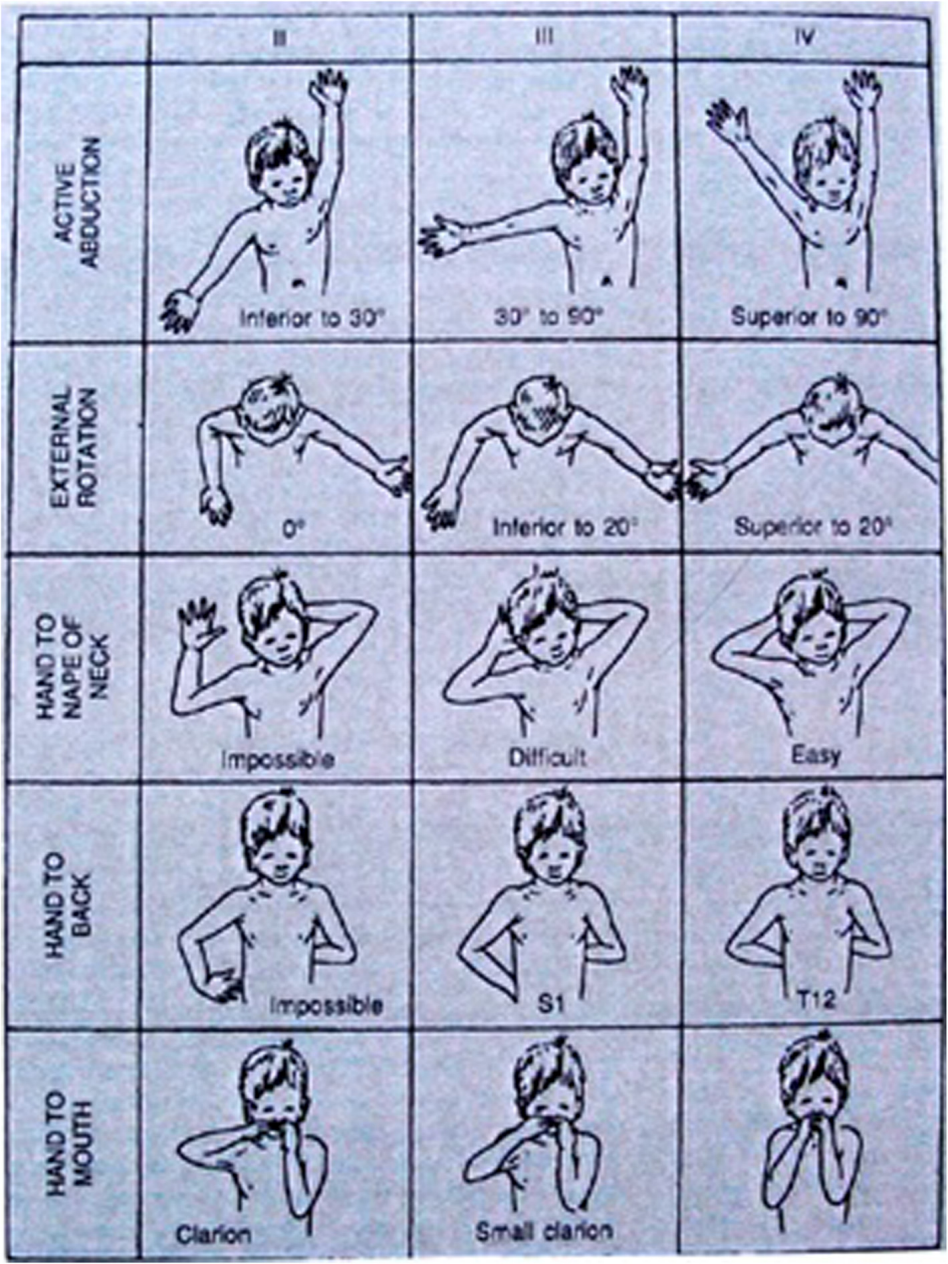
The Mallet score.

This is a score with tests for five functions: 1. shoulder abduction, 2. external rotation with the arm against the side of the body, 3. putting the hand to the nape of the neck, 4. putting the hand on the back as high up as possible and 5. hand touching the mouth. This is not a sliding scale, as 5 grades are recognized: from function not possible (grade 1) to normal function (grade 5). Usually only grades 2, 3 and 4 are depicted in publications (Figure 6). The functions 2, 3 and 5 are tests of external rotation of the shoulder, but only the second function can, if strictly applied, provide the best score for gleno-humeral external rotation. It is important to note that also in this score, the neutral point for the second test is with the hand pointing forward so that grade 2 will give 0° as the result, often interpreted as: “there is no external rotation possible”, which, of course, is not correct as illustrated above.

### How we do it

Measuring active external rotation or any function in the arm may be difficult in a young child who has been pestered because of his brachial plexus lesion by doctors, physiotherapists and other helpful people. It sometimes demands patience from the examiner.

As pointed out in the previous paragraph, the second, third and fifth functions from the Mallet scale are functional tests of external rotation. The second function evaluates external rotation without touching the child and produces an assessment expressed in degrees. Then we examine the third Mallet function. When the child is not able (or only with great difficulty) to put the hand of the affected side in the nape of the neck, we can be sure that the active external rotation is diminished. The same holds true for the fifth Mallet function. When the child in the effort to reach his mouth with his hand, can only succeed by abduction of the arm (the trumpet sign), this also illustrates diminished external rotation (or insufficient strength of the biceps).

We then try to obtain a more accurate measurement expressed in degrees. The patient is asked to stand straight, that is, curvature of the spine to compensate for a lack of external rotation is prevented by the examiner. Additionally, the upper arm is held in adduction by the examiner. Then the patient is asked to rotate his arm externally as far as possible, evoking pure gleno-humeral external rotation, avoiding scapulo-thoracic muscular assistance. Sometimes it is advisable to evoke this movement first in the unaffected arm to familiarize the child with the movement.

In younger children, external rotation is graded by observation of play in three positions: supine, lying on their side and sitting. Attention is attracted using bright toys with rattles and bells with the examiner standing on the affected side. Offering a tit-bit may be helpful. It is evident that accurate measurement is often not possible, because external rotation is examined functionally.

We wish to point out that the insidious problem of fixed deformity, even to a minor degree, will contribute to the problem of loss of active functional movement. The risk of developing a fixed glenohumeral deformity is high whenever the passive external rotation diminishes to under 30° (with the arm held in neutral adduction position).

## Discussion

The positioning of the neutral zero point, more or less in the middle of the range of active external rotation of the shoulder joint, has produced confusing conclusions concerning the gravity of the restriction of active external rotation in case of weakness of the infraspinatus muscle. This has influenced the indications for surgery. Pondaag e.a. [9] reported results on recovery of external rotation after brachial plexus surgery, whether by grafting from C5 or by nerve transfer of the accessory nerve, as disappointingly low, but they did not mention the preoperative results. In our view, their conclusion is based on a misinterpretation of the measurement of external rotation as illustrated above. In their paper, they correctly considered the normal range of external rotation to be −70° to +80 degrees, but they did not specify why they regarded recovery of external rotation as unsatisfactory in their patients. Van Ouwerkerk e.a. [10] showed satisfactory spontaneous recovery of shoulder and hand function in patients with obstetric brachial plexus palsy except for active shoulder external rotation. They thought that a central issue, whether due to defective central learning or due to co-contraction, might be responsible for a disappointing result.

Preoperative electromyographic investigations may often show good voluntary muscle contractions in the infraspinatus muscle. Also many surgeons have found that intraoperative electrical stimulation of the suprascapular nerve shows a good response in the infraspinatus muscle in spite of unsatisfactory function. A central issue may be the cause of co-contraction. Schaakxs e.a. [11], in a series of 65 children who underwent an accessory nerve neurotization, reported that all the patients presented an active external rotation close to 0° (i.e. to the neutral position, meaning 70° only) preoperatively, which they called lack of recovery, disregarding the presence of external rotation from hand–to-abdomen to the neutral zero position. They took samples from the suprascapular nerve from 13 patients for histopathological examination. One patient had a neuroma; the others showed signs of good endoneural regeneration and no signs of degeneration.

## Conclusion

Literature reports of measurements of active joint motion must be regarded with caution. So-called lack of recovery of active rotational movements of the shoulder may not be caused by lack of innervation of the infraspinatus muscle, but by misinterpretation of measurement results. Also, co-contraction or defective central learning may cause insufficient external rotation in spite of sufficient reinnervation of the infraspinatus muscle. Co-contraction causing defective external rotation can be registered with multi-channel electromyography and it can (possibly) be treated by Botox-injection into the subscapularis muscle.

## Competing interests

The authors declare that they have no competing interests.

## Authors’ contributions

GB and RM contributed equally to this paper. Both authors read and approved the final manuscript.
